# Supplementary material for: Quantitative live-imaging reveals the dynamics of apical cells during gametophyte development in ferns
Source: Quant Plant Biol. 2022 Nov 17;3:e25. doi: 10.1017/qpb.2022.21 (PMC10095955; doi:10.1017/qpb.2022.21)
Supplement: Supplementary file 1 [file qpbsup.zip › S2632882822000212sup001.pdf]

Supplementary files

Supplementary Figures S1-S14

Supplementary Tables S1-S18

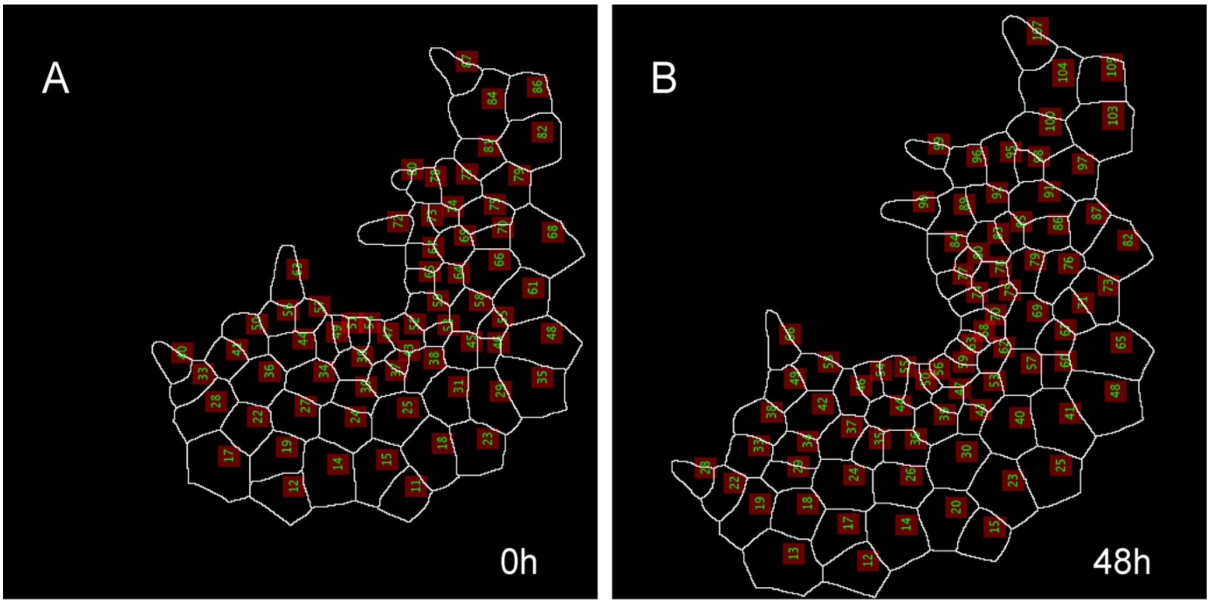

Fig. S1. Computational segmentation and quantification of the confocal images in Figure 2A-E. (A) shows each segmented cell with a unique ID from a gametophyte imaged at 0 h (shown in Figure 2A). (B) shows each segmented cell with a unique ID from the gametophyte imaged at 48 h (shown in Figure 2C). The quantified area of each segmented cell with the ID (A-B) is included in Supplementary Tables S1-S2.

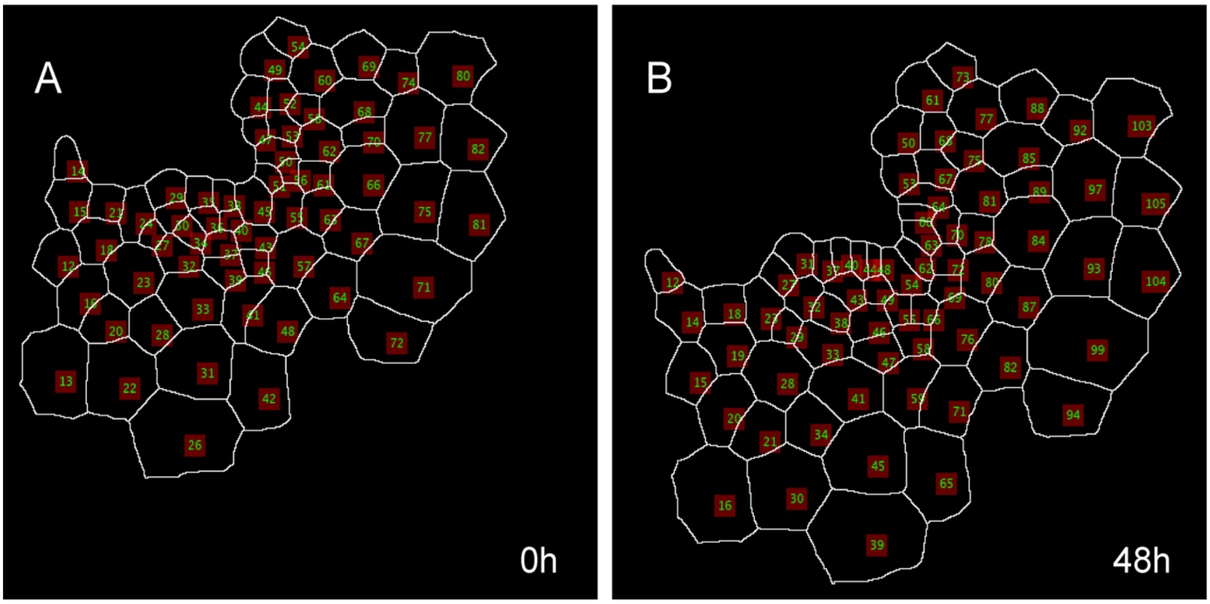

Fig. S2. Computational segmentation and quantification of the confocal images in Figure 2F-J. (A) shows each segmented cell with a unique ID from a gametophyte imaged at 0 h (shown in Figure 2F). (B) shows each segmented cell with a unique ID from the gametophyte imaged at 48 h (shown in Figure 2H). The quantified area of each segmented cell with the ID (A-B) is included in Supplementary Tables S3-S4.

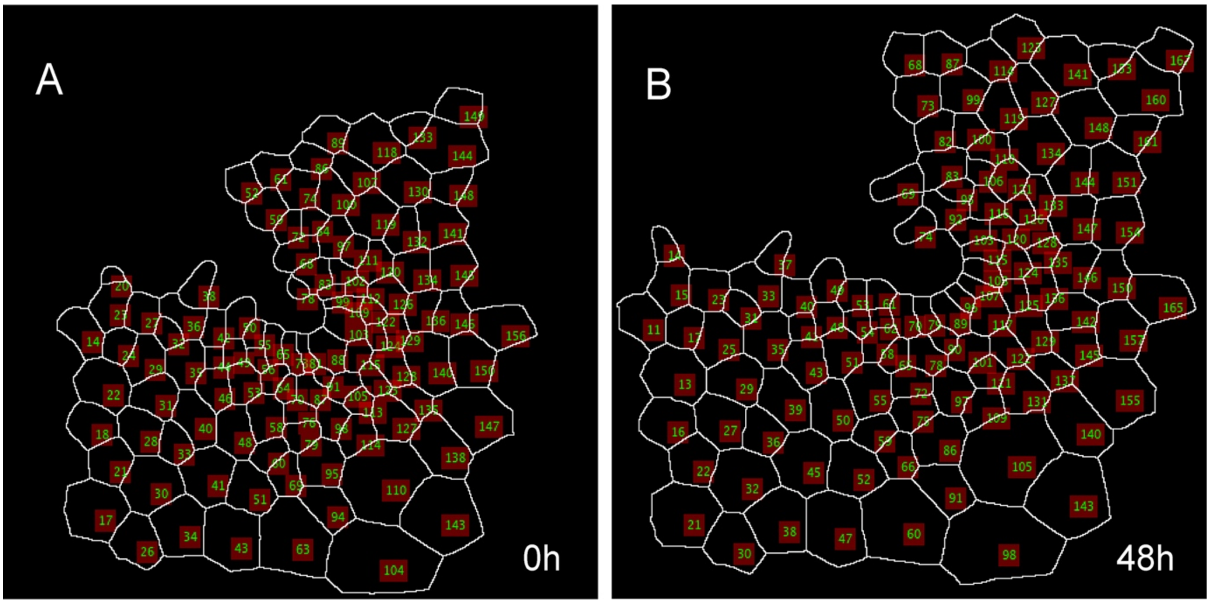

Fig. S3. Computational segmentation and quantification of the confocal images in Figure 3A1-A5. (A) shows each segmented cell with a unique ID from a gametophyte imaged at 0 h (shown in Figure 3A1). (B) shows each segmented cell with a unique ID from the gametophyte imaged at 48 h (shown in Figure 3A3). The quantified area of each segmented cell with the ID (A-B) is included in Supplementary Tables S5-S6.

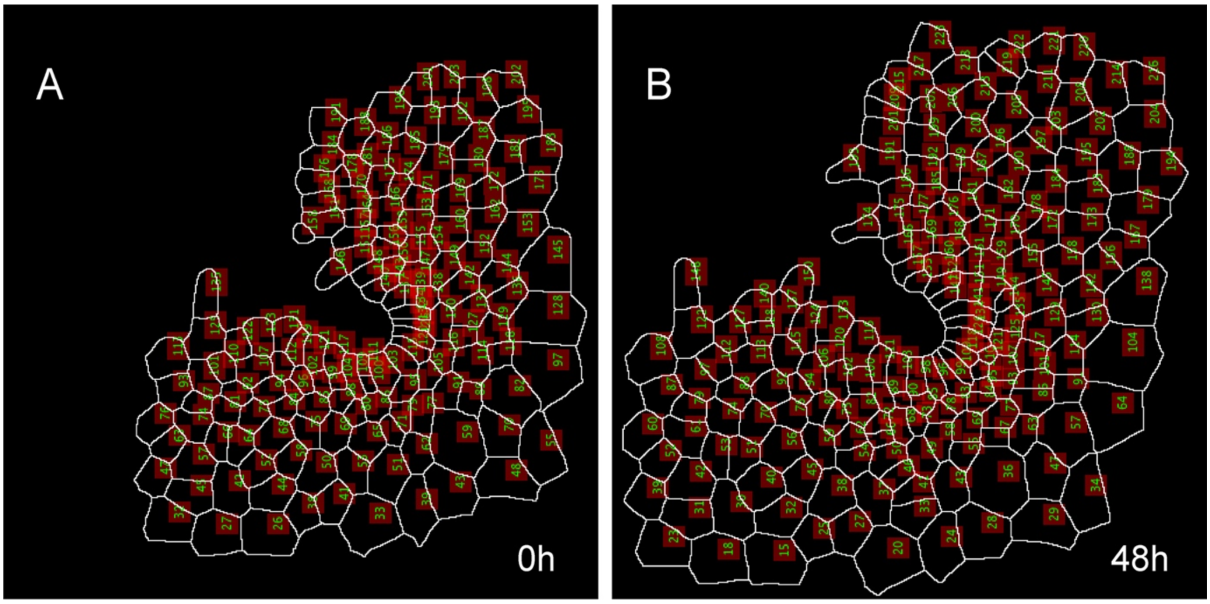

Fig. S4. Computational segmentation and quantification of the confocal images in Figure 3B1-B5. (A) each segmented cell with a unique ID from a gametophyte imaged at 0 h (Figure 3B1). (B) shows each segmented cell with a unique ID from the gametophyte imaged at 48 h (shown in Figure 3B3). The quantified area of each segmented cell with the ID (A-B) is shown in Supplementary Tables S7-S8.

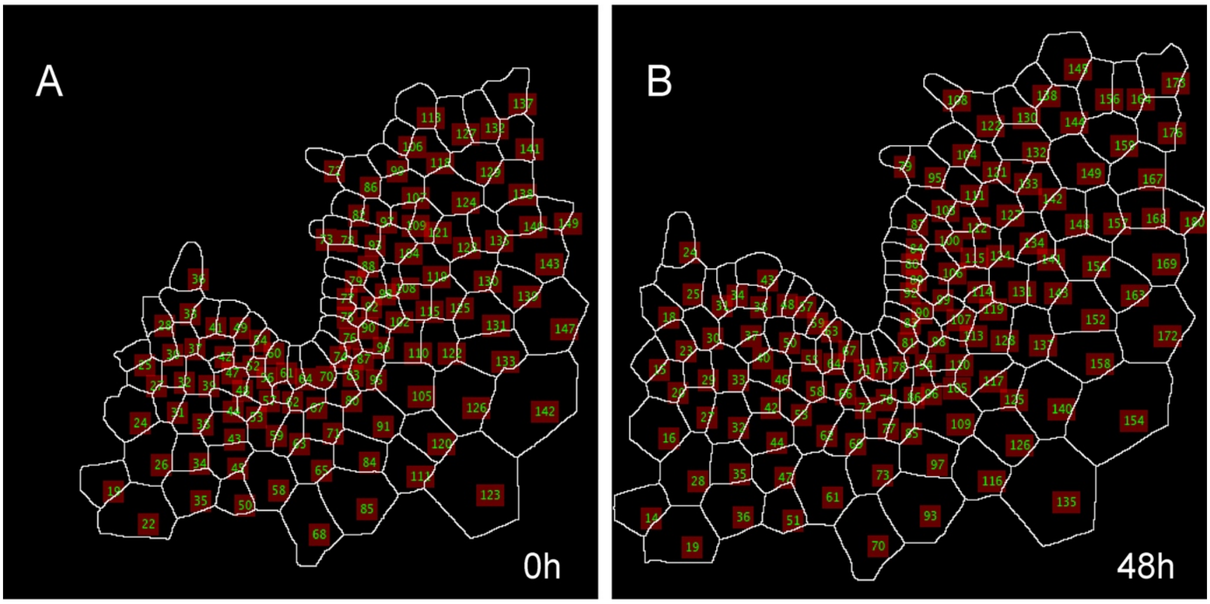

Fig. S5. Computational segmentation and quantification of the confocal images in Figure 3C1-C5. (A) shows each segmented cell with a unique ID from a gametophyte imaged at 0 h (shown in Figure 3C1). (B) shows each segmented cell with a unique ID from the gametophyte imaged at 48 h (shown in Figure 3C3). The quantified area of each segmented cell with the ID (A-B) is shown in Supplementary Tables S9-S10.

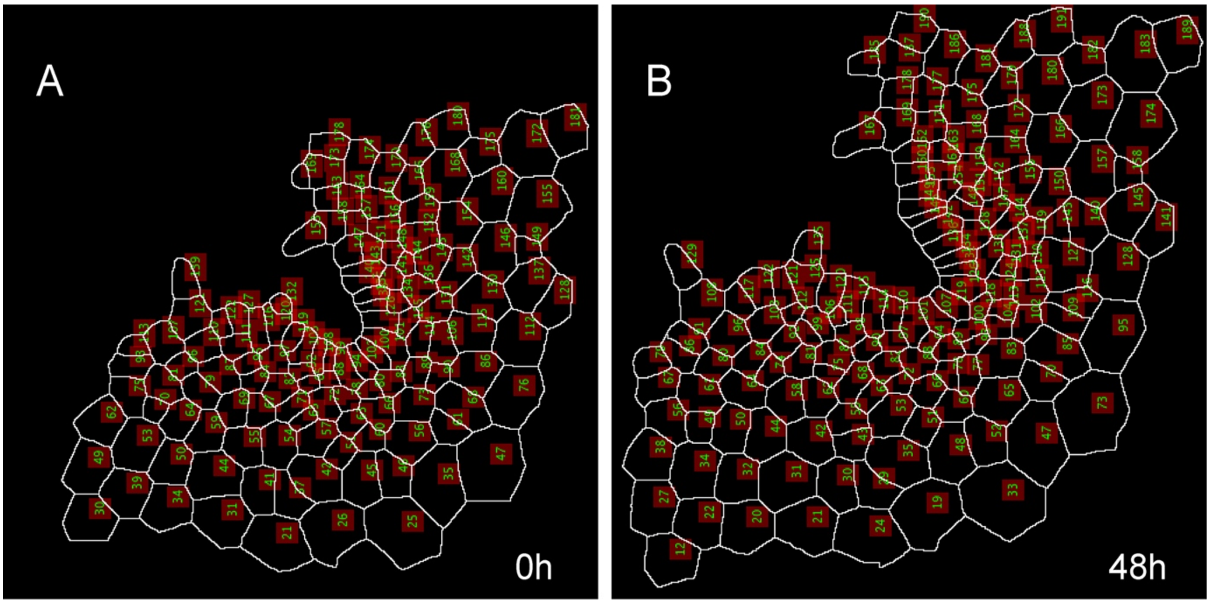

Fig. S6. Computational segmentation and quantification of the confocal images in Figure 3D1-D5. (A) shows each segmented cell with a unique ID from a gametophyte imaged at 0 h (shown in Figure 3D1). (B) shows each segmented cell with a unique ID from the gametophyte imaged at 48 h (shown in Figure 3D3). The quantified area of each segmented cell with the ID (A-B) is shown in Supplementary Tables S11-S12.

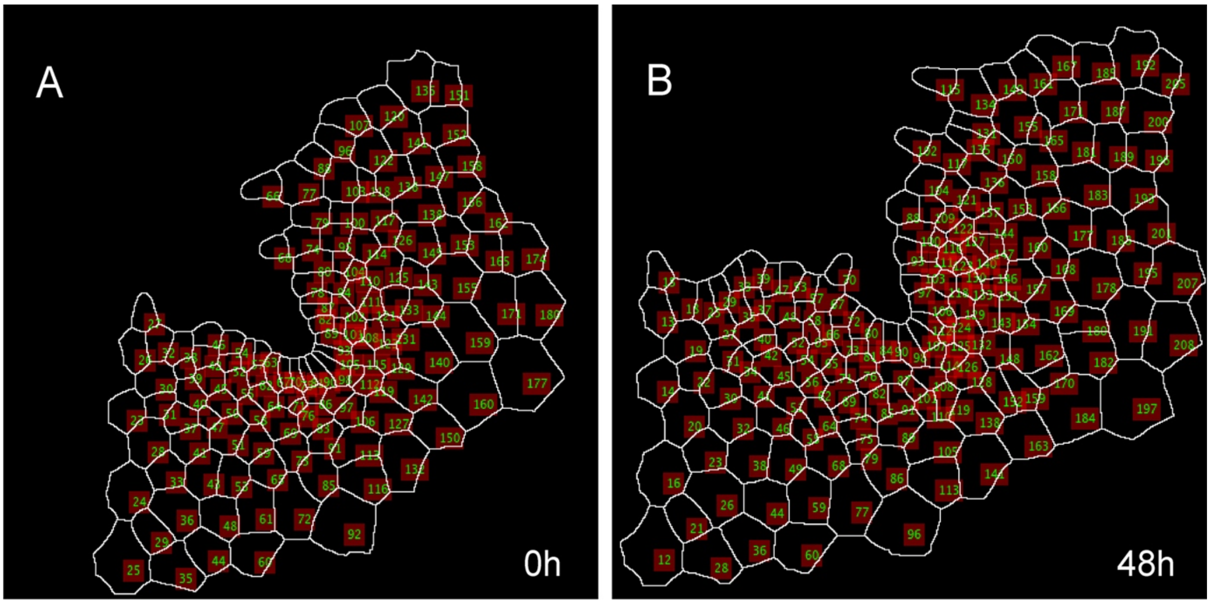

Fig. S7. Computational segmentation and quantification of the confocal images in Figure 3E1-E5. (A) shows each segmented cell with a unique ID from a gametophyte imaged at 0 h (shown in Figure 3E1). (B) shows each segmented cell with a unique ID from the gametophyte imaged at 48 h (shown in Figure 3E3). The quantified area of each segmented cell with the ID (A-B) is shown in Supplementary Tables S13-S14.

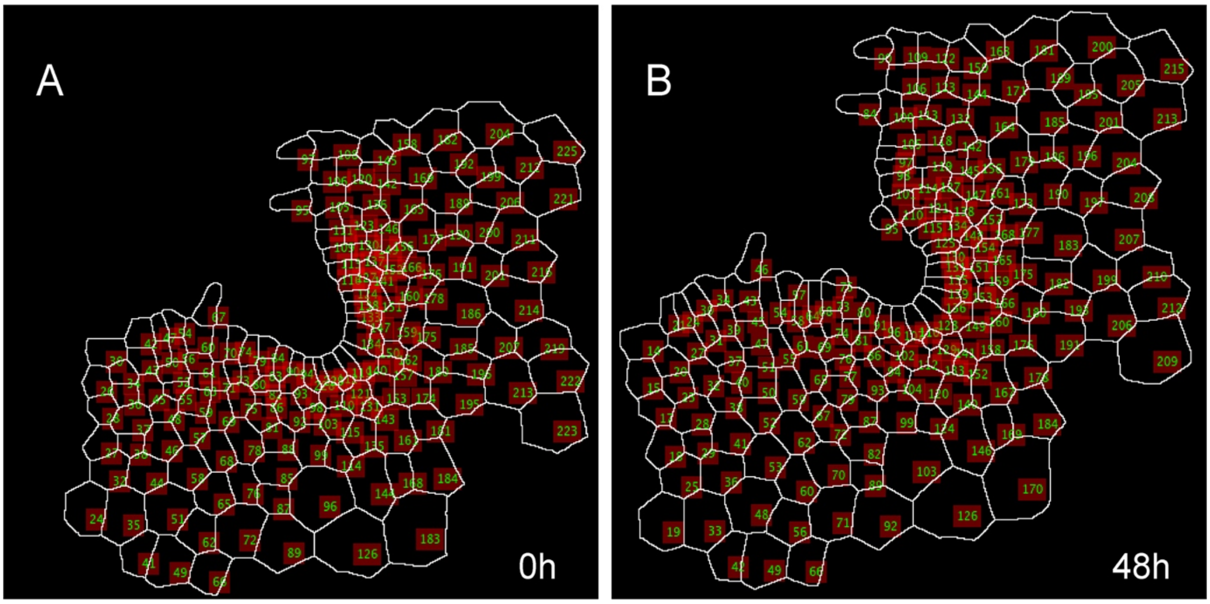

Fig. S8. Computational segmentation and quantification of the confocal images in Figure 3F1-F5. (A) shows each segmented cell with a unique ID from a gametophyte imaged at 0 h (shown in Figure 3F1). (B) shows each segmented cell with a unique ID from the gametophyte imaged at 48 h (shown in Figure 3F3). The quantified area of each segmented cell with the ID from (A-B) is shown in Supplementary Tables S15-S16.

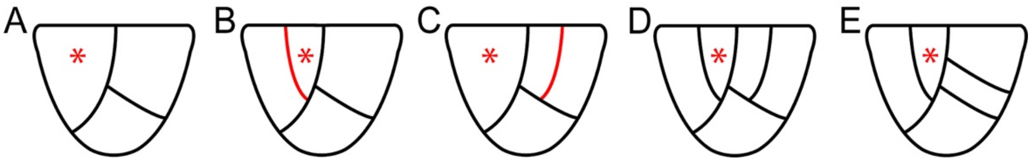

Fig. S9. Diagrams and illustration of cell division patterns in the AC and its immediate progenies. (A-E) The diagrams illustrate the conserved cell packets composed of the wedge-shaped AC (indicated by a red star) and its immediate progenies. Red stars indicate the wedge-shaped apical cells. At least three independent biological replicates showed comparable division patterns that led to the cell packets illustrated in (D) and (E) over 48 hours, respectively. The suggested new wall formation was labeled in red.

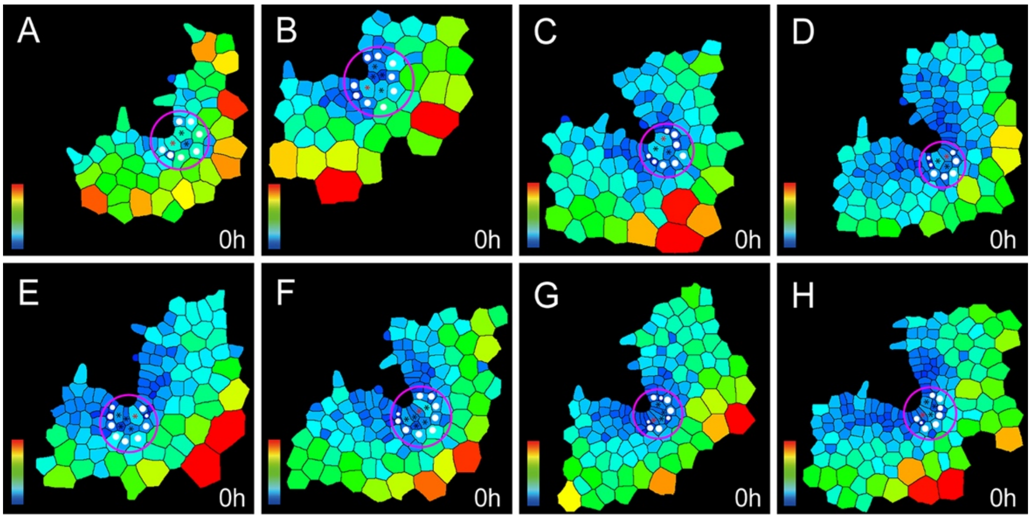

Fig. S10. Cells at the center of the meristem and outside the center in the *Sphenomeris chinensis* gametophytes at 0 h. (A-H) Purple circles highlighted the center of the meristem of each gametophyte at 0 h as the center group for area quantification (shown in Figures 4A, B, C, D, E, F, G, and H), respectively. The center of the meristem at 0 h included the conserved cell packet composed of the wedge-shaped AC (indicated by a red star) and its adjacent progenies (indicated by black stars). The center of the meristem also included the cells surrounding the star-labeled cells (indicated by white dots). All the other segmented cells (except trichomes) in each gametophyte (A, B, C, D, E, F, G, H) were included in the quantification as outside of the center group (Figure 4A, B, C, D, E, F, G, and H). The eight samples (A-H) were also analyzed and shown in Figures 2B, 2G, 3A2, 3B2, 3C2, 3D2, 3E2, and 3F2, respectively.

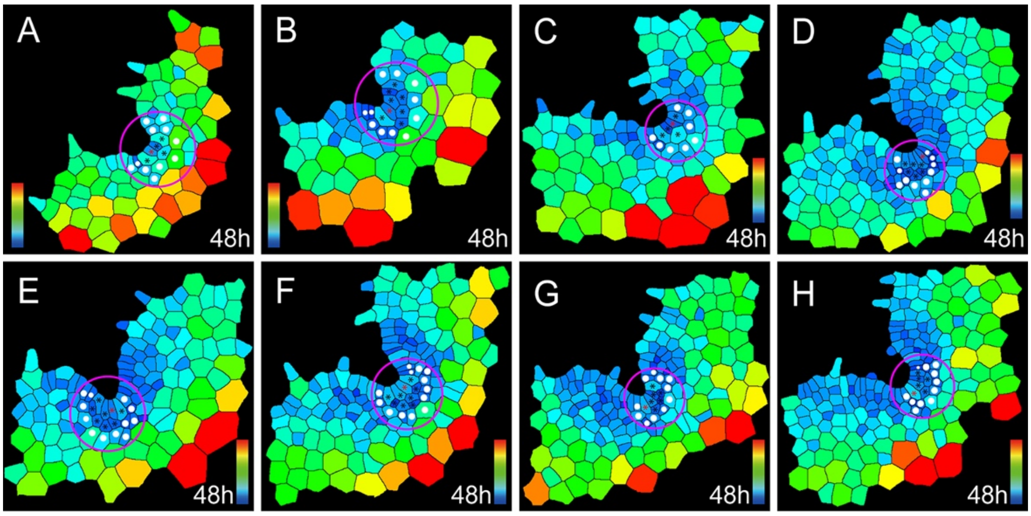

Fig. S11. Cell progenies at the center of the meristem and outside the center in the *Sphenomeris chinensis* gametophytes at 48 h. (A-H) Purple circles highlighted the center of the meristem for each gametophyte at 48 h, which was included as the center group for area quantification (Supplementary Figure S12A-H), respectively. The center of the meristem at 48 h included all the progenies of cells at the center of the meristem at 0 h, including progenies of the cells from the cell packet (indicated by stars) and progenies of the cells surrounding the star-labeled cell packet (indicated by white dots) (Supplementary Figure S10A, B, C, D, E, F, G, H). The wedge-shaped ACs (A-H) were indicated by red stars. All the other segmented cells (except trichomes) in (A, B, C, D, E, F, G, H) were included in the quantification as the outside of the center (Supplementary Figure S12A-H), respectively. The eight samples (A-H) were also analyzed and shown in Figures 2D, 2I, 3A4, 3B4, 3C4, 3D4, 3E4, and 3F4, respectively.

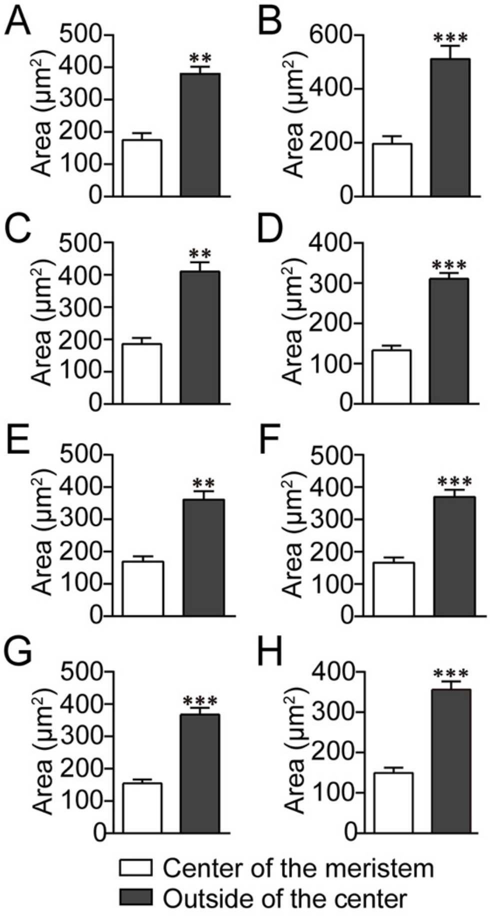

Fig. S12. Cell size variation in the meristems and gametophytes at 48 h. (A-H) show the area quantification of the labeled cells from each gametophyte (Supplementary Figures S11A-H), respectively. (A-H) Y-axis showed the average cell area, and X-axis represented the center of the meristem (white) and outside of the center (black). (A) *n*= 16 cells at the center of the meristem and 57 cells outside the center. (B) *n*= 19 cells at the center of the meristem and 49 cells outside the center. (C) *n*= 13 cells from the center of the meristem and 95 cells outside the center. (D) *n*= 18 cells at the center of the meristem and 157 cells outside the center. (E) *n*= 20 cells at the center of the meristem and 101 cells outside the center. (F) *n*= 22 cells at the center of the meristem and 125 cells outside the center. (G) *n*= 18 cells at the center of the meristem and 143 cells outside the center. (H) *n*= 17 cells at the center of the meristem and 152 cells outside the center. Bars: means ± standard errors. ** $P < 0.01$, *** $P < 0.001$ (Student's two-tailed t-test).

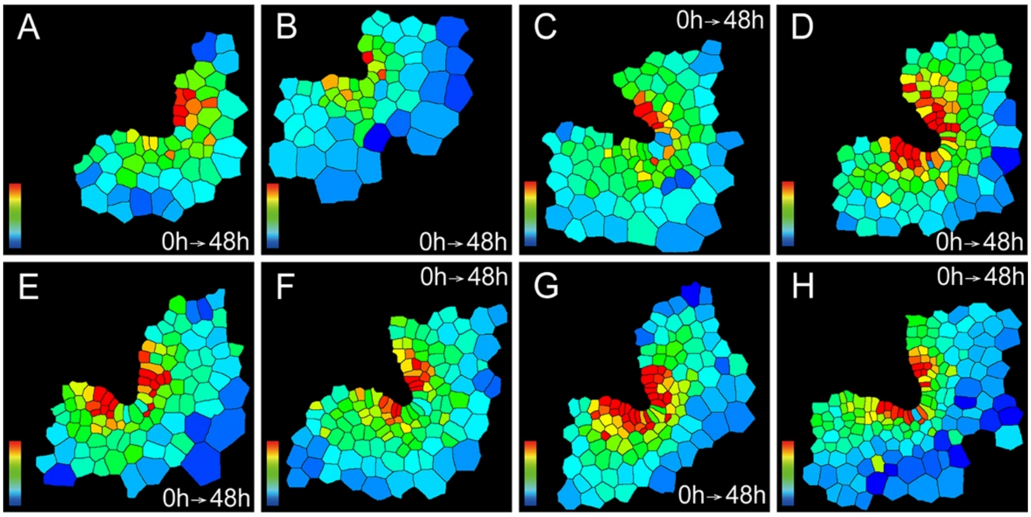

Fig. S13. Quantification and visualization of the relative cell growth in gametophytes over the 48 hours. The relative growth of each cell = [the cell area (including the area of its progenies) at 48 h – the cell area at 0 h] / the cell area at 0 h. Color bars (A-H) quantitatively indicated the scale for the relative cell growth, ranging from blue (0) to red (≥1). The eight samples (A-H) were also analyzed and shown in Figures 2B, 2G, 3A2, 3B2, 3C2, 3D2, 3E2, and 3F2.

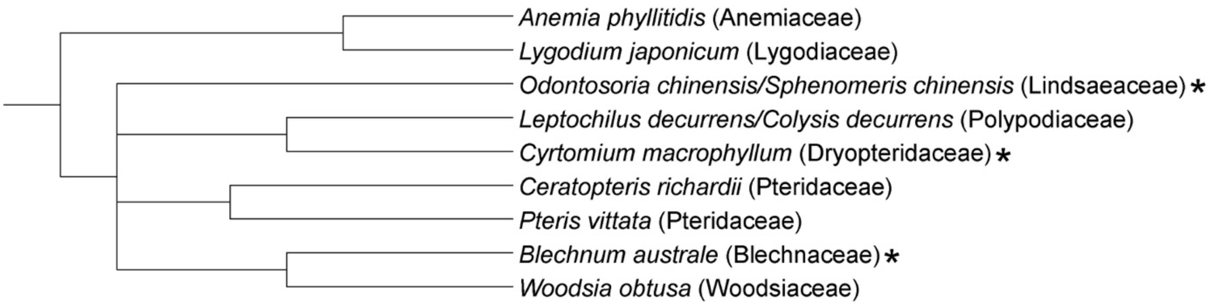

Fig. S14. The phylogenetic tree of nine fern species. The phylogenetic tree was generated using PHYLOT (https://phylot.biobyte.de/index.cgi). Stars indicate the fern species with persistent apical cells in the gametophytes.

Table S1. Area quantification of each segmented cell from the *Sphenomeris chinensis* gametophyte at 0 h (Figure 2A-B and Supplementary Figure S1A).

Table S2. Area quantification of each segmented cell from the *Sphenomeris chinensis* gametophyte at 48 h (Figure 2C-D and Supplementary Figure S1B).

Table S3. Area quantification of each segmented cell from the *Sphenomeris chinensis* gametophyte at 0 h (Figure 2F-G and Supplementary Figure S2A).

Table S4. Area quantification of each segmented cell from the *Sphenomeris chinensis* gametophyte at 48 h (Figure 2H-I and Supplementary Figure S2B).

Table S5. Area quantification of each segmented cell from the *Sphenomeris chinensis* gametophyte at 0 h (Figure 3A1-A2 and Supplementary Figure S3A).

Table S6. Area quantification of each segmented cell from the *Sphenomeris chinensis* gametophyte at 48 h (Figure 3A3-A4 and Supplementary Figure S3B).

Table S7. Area quantification of each segmented cell from the *Sphenomeris chinensis* gametophyte at 0 h (Figure 3B1-B2 and Supplementary Figure S4A).

Table S8. Area quantification of each segmented cell from the *Sphenomeris chinensis* gametophyte at 48 h (Figure 3B3-B4 and Supplementary Figure S4B).

Table S9. Area quantification of each segmented cell from the *Sphenomeris chinensis* gametophyte at 0 h (Figure 3C1-C2 and Supplementary Figure S5A).

Table S10. Area quantification of each segmented cell from the *Sphenomeris chinensis* gametophyte at 48 h (Figure 3C3-C4 and Supplementary Figure S5B).

Table S11. Area quantification of each segmented cell from the *Sphenomeris chinensis* gametophyte at 0 h (Figure 3D1-D2 and Supplementary Figure S6A).

Table S12. Area quantification of each segmented cell from the *Sphenomeris chinensis* gametophyte at 48 h (Figure 3D3-D4 and Supplementary Figure S6B).

Table S13. Area quantification of each segmented cell from the *Sphenomeris chinensis* gametophyte at 0 h (Figure 3E1-E2 and Supplementary Figure S7A).

Table S14. Area quantification of each segmented cell from the *Sphenomeris chinensis* gametophyte at 48 h (Figure 3E3-E4 and Supplementary Figure S7B).

Table S15. Area quantification of each segmented cell from the *Sphenomeris chinensis* gametophyte at 0 h (Figure 3F1-F2 and Supplementary Figure S8A).

Table S16. Area quantification of each segmented cell from the *Sphenomeris chinensis* gametophyte at 48 h (Figure 3F3-F4 and Supplementary Figure S8B).

Table S17. Summary of cell division activity at the center of the meristem or outside the center in the *Sphenomeris chinensis* gametophytes.

Table S18. Summary of cell area increases at the center of the meristem or outside the center in the *Sphenomeris chinensis* gametophytes.
